# Nerve Ultrasound Score in Chronic Inflammatory Demyelinating Polyneuropathy

**DOI:** 10.3390/medicina59040747

**Published:** 2023-04-11

**Authors:** Cheng-Yin Tan, Mohd Azly Yahya, Khean-Jin Goh, Nortina Shahrizaila

**Affiliations:** 1 Division of Neurology, Department of Medicine, University of Malaya, Kuala Lumpur 50603, Malaysianortina@um.edu.my (N.S.); 2 Neurophysiology Laboratory, University of Malaya Medical Centre, Kuala Lumpur 59100, Malaysia

**Keywords:** acute inflammatory demyelinating polyneuropathy, chronic inflammatory demyelinating polyneuropathy, Guillain-Barré syndrome, nerve ultrasound, ultrasound pattern sum score

## Abstract

*Background and Objectives*: Studies have suggested that, by applying certain nerve ultrasound scores, demyelinating and axonal polyneuropathies can be differentiated. In the current study, we investigated the utility of ultrasound pattern sub-score A (UPSA) and intra- and internerve cross-sectional area (CSA) variability in the diagnostic evaluation of demyelinating neuropathies. *Materials and Methods*: Nerve ultrasound was performed in patients with chronic inflammatory demyelinating polyneuropathy (CIDP) and acute inflammatory demyelinating polyneuropathy (AIDP) and compared to patients with axonal neuropathies. The UPSA, i.e., the sum of ultrasound scores at eight predefined measurement points in the median (forearm, elbow and mid-arm), ulnar (forearm and mid-arm), tibial (popliteal fossa and ankle) and fibular (lateral popliteal fossa) nerves, was applied. Intra- and internerve CSA variability were defined as maximal CSA/minimal CSA for each nerve and each subject, respectively. *Results*: A total of 34 CIDP, 15 AIDP and 16 axonal neuropathies (including eight axonal Guillain-Barré syndrome (GBS), four hereditary transthyretin amyloidosis, three diabetic polyneuropathy and one vasculitic neuropathy) were included. A total of 30 age- and sex-matched healthy controls were recruited for comparison. Significantly enlarged nerve CSA was observed in CIDP and AIDP with significantly higher UPSA in CIDP compared to the other groups (9.9 ± 2.9 vs. 5.9 ± 2.0 vs. 4.6 ± 1.9 in AIDP vs. axonal neuropathies, *p* < 0.001). A total of 89.3% of the patients with CIDP had an UPSA score ≥7 compared to the patients with AIDP (33.3%) and axonal neuropathies (25.0%) (*p* < 0.001). Using this cut-off, the performance of UPSA in differentiating CIDP from other neuropathies including AIDP was excellent (area under the curve of 0.943) with high sensitivity (89.3%), specificity (85.2%) and positive predictive value (73.5%). There were no significant differences in intra- and internerve CSA variability between the three groups. *Conclusion*: The UPSA ultrasound score was useful in distinguishing CIDP from other neuropathies compared to nerve CSA alone.

## 1. Introduction

There is growing interest in the complementary use of nerve ultrasound in the evaluation of peripheral neuropathies. Nerve ultrasound can help differentiate demyelinating polyneuropathies from axonal neuropathies [1,2,3,4]. In previous studies of patients with chronic inflammatory demyelinating polyneuropathy (CIDP), nerve ultrasound demonstrated either multifocal or generalised enlargement of the nerve cross-sectional area (CSA) [3,5].

Recent studies have also suggested that, by applying standardised scores such as ultrasound pattern sum score (UPSS) and Bochum ultrasound score (BUS) to quantify nerve enlargement [6,7,8], the different polyneuropathies can be better distinguished. Both scores were increased in demyelinating neuropathies compared to axonal neuropathies. In one study, the subscore-A of the ultrasound pattern sum score (UPSS), also known as UPS-A, was reported to be sensitive and specific in differentiating CIDP from acute inflammatory demyelinating polyneuropathy (AIDP) and other neuropathies, including vasculitic neuropathy, critical illness polyneuropathy and toxic and metabolic neuropathies [7].

In CIDP, ultrasound may show heterogeneous nerve enlargement with the CSA predominantly increased in proximal nerve segments and non-entrapment sites [8,9,10]. A few studies have described several novel ultrasound measures, including intranerve and internerve CSA variability, aiming to quantify cross-sectional changes in peripheral nerves as the new sonographic approach [11,12]. The introduction of intranerve and internerve CSA variability measures highlights the heterogeneous and multifocal enlargement of nerves in immune-mediated neuropathies.

In the current study, we aimed to evaluate the utility of the UPSA and intra- and internerve CSA variability in differentiating demyelinating neuropathies (CIDP and AIDP) from various axonal neuropathies. We hypothesise that the UPSA scores and intra- and internerve CSA variability are higher in demyelinating neuropathies, specifically in CIDP, as compared to axonal neuropathies.

## 2. Materials and Methods

### 2.1. Subjects

Consecutive patients with CIDP and Guillain-Barré syndrome (GBS) presenting to the University of Malaya Medical Centre (UMMC), Kuala Lumpur from January 2016 to June 2022 were included in the study. The diagnosis of CIDP and GBS were made based on the European Academy of Neurology and Peripheral Nerve Society (EAN/PNS) 2021 criteria and National Institute of Neurological Disorders and Stroke (NINDS) criteria, respectively [13,14]. Within the same period, patients with axonal neuropathies who were observed in our neurophysiology laboratory for nerve conduction studies (NCS) were invited to participate in the study. Patients with normal or equivocal electrophysiology were excluded from the study [13,15,16]. The control group consisted of healthy volunteers with no prior history of neurological disorders. Demographic data, including height, weight and body mass index (BMI), were collected at the time of the study. Disease duration was calculated based on the time of disease onset. Nerve ultrasound and NCS were performed on all participants. The study was approved by the Medical Research Ethics Committee of UMMC (201697-4225). Written informed consent was obtained from all participants.

### 2.2. Nerve Ultrasound

Nerves were scanned as previously described using a Mindray^®^ M7 ultrasound machine with the linear array transducer set at a frequency of 8 to 12 MHz [17] by two evaluators (C.Y.T. and M.A.Y.) with at least 5 years of experience in neuromuscular ultrasound and who were not blinded to the clinical and neurophysiologic data of the patients. In brief, the nerve ultrasound was performed on both sides at predefined segments of the following nerves: median and ulnar nerves at the distal wrist crease, forearm (10 cm proximal to the distal wrist crease), elbow (antecubital fossa for the median nerve and at the level of the medial epicondyle for the ulnar nerve) and mid arm (8 cm above the elbow); superficial radial nerve at the mid forearm (midpoint between the wrist and elbow); posterior tibial nerve at the popliteal fossa and posterior to the medial malleolus at the ankle; fibular nerve at the fibular head and lateral popliteal fossa; and sural nerve at the Achilles tendon between the two heads of the gastrocnemius, 10 cm above the lateral malleolus. The nerve CSA was measured with the tracing inside the hyperechoic rim of each nerve.

### 2.3. Quantification of Ultrasound Scores

Where applicable, the intranerve, internerve and side-to-side difference ratio of the intranerve CSA variability (SSDIVA) were calculated as follows: (1) the intranerve CSA variability (for each nerve) defined as maximal CSA/minimal CSA, (2) the internerve CSA variability (for each subject) defined as maximal intranerve CSA variability/minimal intranerve CSA variability, and (3) the side-to-side difference ratio of the intranerve CSA variability (for each nerve) defined as side with maximal intranerve CSA variability/side with minimal intranerve CSA variability [11,12].

**Figure 1:**
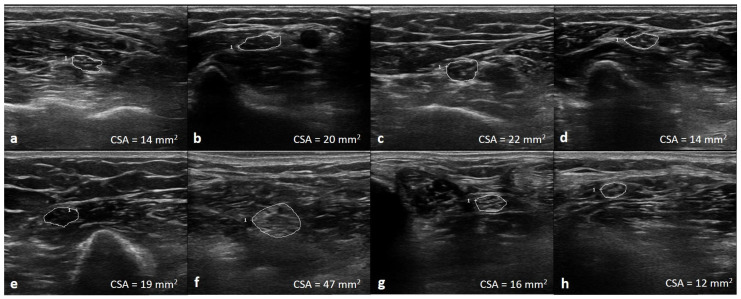
Illustration of the ultrasonographic nerve segments at median forearm (**a**), elbow (**b**), mid-arm (**c**); ulnar forearm (**d**), mid-arm (**e**); tibial knee (**f**), ankle (**g**); and fibular knee (**h**) of one of the CIDP patients. Total UPSA score: (2 + 2 + 2 + 2 + 2 + 2 + 1 + 1) = 14.

The UPSA was calculated as follows: median nerve CSA at the mid arm, elbow and forearm; ulnar nerve CSA at the mid arm and forearm; tibial nerve CSA at the popliteal fossa and ankle; and fibular nerve CSA at the lateral popliteal fossa (Figure 1). Each CSA value of the peripheral nerves at the anatomical locations described above was evaluated according to previous published boundary values [18]. Individual nerve enlargement >100% and <150% of the referenced upper limit of normal was scored 1, and individual nerve CSA >150% was scored 2. The maximum score was 16 (Supplementary Materials).

### 2.4. Nerve Conduction Studies

Nerve conduction studies (NCS) were performed by C.Y.T. as previously described using the Medelec^TM^ Synergy Electromyography machine [19]. Sensory studies were performed in the median, ulnar, radial and sural nerves. Motor studies were carried out on the median, ulnar, fibular and tibial nerves. The distal motor latency, motor conduction velocity, compound muscle action potential (CMAP) amplitude and F-wave latency were measured. Similarly, sensory nerve action potential (SNAP) amplitude and sensory conduction velocity were obtained. Reference values were derived from previously established normal ranges at our laboratory [20].

### 2.5. Statistical Analysis

Statistical analyses were performed with SPSS version 24.0 (IBM corp, New York, NY, USA). Only the nerve CSA values obtained from the right side were used for the analysis, except for the calculation of SSDIVA. The inclusion of values from both sides for each subject would artificially lower the variance. Comparisons between the different groups were made for CIDP, AIDP, axonal neuropathies and healthy controls. Categorical data were presented as numbers and percentages and compared with the Chi-square test. Continuous data were presented as the mean ± standard deviation (SD) and compared with the ANOVA test. Bonferroni correction was applied for multiple comparisons to reduce the Type 1 error with a more stringent *p* value of <0.0125 (α/n) considered statistically significant. Performance of the UPSA in determining CIDP was assessed with the area under the receiver operating curve (AUC). The sensitivity, specificity and positive predictive value of the UPSA in differentiating CIDP from other neuropathies and controls were calculated from a 2 × 2 contingency table.

## 3. Results

A total of 34 patients with CIDP were included in the study. All patients fulfilled the EAN/PNS 2021 clinical criteria. Of the 34 patients, 28 were typical CIDP, five were distal CIDP, and one was multifocal CIDP. A total of 30 patients with GBS who fulfilled the NINDS criteria were recruited within the same period with 23 classical GBS and seven Miller Fisher syndrome. Among the patients with CIDP, all but one fulfilled the current demyelinating electrodiagnostic criteria (Supplementary Materials). In one patient, the nerves were inexcitable, fulfilling the possible CIDP diagnostic category with supportive criteria. In the GBS group, 15 patients were AIDP, eight were axonal, five were equivocal, and two had normal electrophysiology based on the Uncini et al. criteria [16]. The latter two groups were excluded from the analysis in the current study. A further eight patients with axonal neuropathies were recruited as follows: hereditary transthyretin amyloidosis (4), diabetic polyneuropathy (3) and vasculitic neuropathy (1). Diagnoses of the latter group of axonal neuropathies were made based on the clinical features, laboratory testing and NCS. In total, there were 16 patients with axonal neuropathies (Table 1). A total of 30 healthy participants were recruited as controls during the study period. The baseline demographics and characteristics are shown in Table 1. There were no differences in the mean age, gender, weight, height and BMI between the four groups. The disease duration for the patients with CIDP was significantly longer than the AIDP group (100.6 ± 116.3 vs. 3.6 ± 2.6 weeks, *p* = 0.004).

There were significant differences in CSA values between the four groups (ANOVA, *p* < 0.0125) (Table 2), except the fibular nerve at the fibular head. The CSA values were significantly larger in the CIDP group compared to the healthy controls at all nerve segments (*p* < 0.0125) (Table 3). The nerve CSAs were also significantly larger when compared to axonal neuropathies for the median and ulnar nerves (except at the entrapment sites) (*p* < 0.0125). In comparison to AIDP, only the CSAs of the median nerve at the elbow and mid arm of the patients with CIDP were significantly larger (*p* < 0.0125).

The CSAs of the patients with AIDP were significantly larger than the controls in the median nerve at the wrist and forearm, ulnar nerve at the wrist and mid arm, tibial nerve at the popliteal fossa and superficial radial nerve at the forearm (*p* < 0.0125). However, there were no significant differences in the nerve CSAs between the AIDP and the axonal neuropathies groups. The median and ulnar intra- and internerve CSA variability as well as the SSDIVA were not significantly different between all groups.

**Figure 2:**
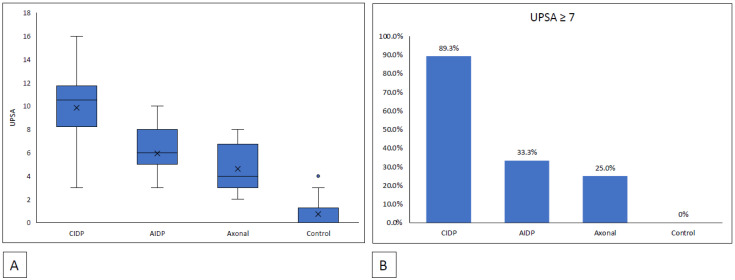
(**A**) Box plots of the mean UPSA scores for CIDP, AIDP, axonal neuropathies and healthy controls. (**B**) Proportion of the groups with UPSA scores of ≥7.

The UPSA scores were significantly higher in the CIDP group (9.9 ± 2.9) compared to the AIDP group (5.9 ± 2.0), axonal neuropathies group (4.6 ± 1.9) and controls (0.7 ± 1.1) (*p* < 0.001) (Figure 2A). For the AIDP group, the UPSA scores were significantly higher when compared to the controls (*p* < 0.001) but not to the axonal neuropathies group (*p* = 0.321). However, 89.3% (25/28) of the patients with CIDP had UPSA scores of at least 7 points compared to the patients with AIDP (33.3%, 5/15) (*p* < 0.001) (Figure 2B).

**Figure 3:**
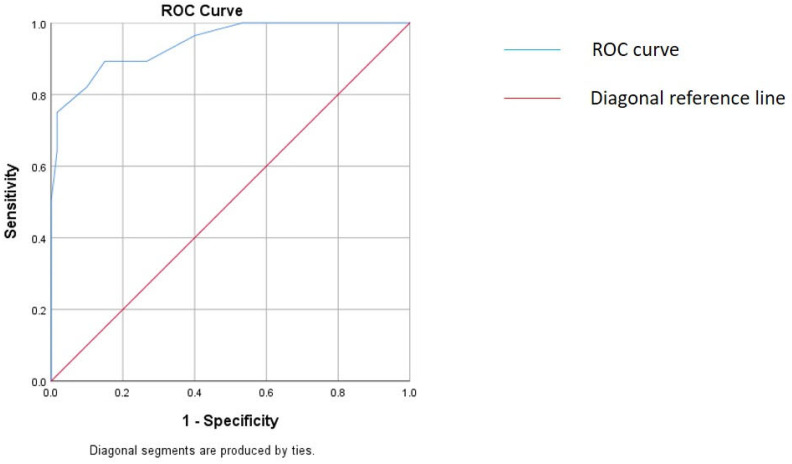
ROC curve analysis of the UPSA score in differentiating CIDP from other neuropathies and controls (AUC = 0.943, *p* < 0.001).

The utility of UPSA in differentiating CIDP from other neuropathies and controls was excellent with an AUC of 0.943 (Table 4, Figure 3). With the cut-off of UPSA ≥ 7, the sensitivity was 89.3%, specificity was 85.2%, and positive predictive value (PPV) was 73.5%.

## 4. Discussion

In the current study, we found significantly higher CSA values of peripheral nerves in CIDP. These findings are in keeping with previous reports [3,5]. Patients with CIDP and AIDP had significantly larger nerves compared to healthy controls, although the nerves were significantly larger in CIDP compared to AIDP.

Studies have suggested that nerves are enlarged during the early stages of CIDP, which worsens over time [21]. In GBS, these changes improve over a period of time with nerve size returning to baseline with recovery [22]. This is in contrast to CIDP where the nerve remains enlarged [23].

The patterns of nerve enlargement differ between CIDP and AIDP. In CIDP, the changes are homogeneous, whereas in AIDP, the changes are patchy. In CIDP, there is a predisposition to enlargement of the nerves in the upper limbs and cervical roots as opposed to AIDP, which tends to involve the nerve roots and the vagus nerve [6,7,8,10,21,24]. The nerve enlargement in CIDP results from both primary demyelination and recurrent episodes of demyelination and remyelination causing oedema [21]. With on-going inflammation, the pattern of nerve enlargement is homogenous as opposed to acute inflammatory neuropathies, which are monophasic events that cause more regional nerve enlargement [21]. In CIDP, there is also a correlation between nerve enlargement and disease duration [21,23].

Previous studies have demonstrated that the UPSA score, which looks at the sum of several nerves, could support a diagnosis of CIDP. Using a cut-off UPSA score of ≥7 points, the AUC was 0.950 with a sensitivity of 80%, specificity of 93% and PPV of 80% (Table 4) [7]. In the current study, the UPSA scores were increased in both AIDP and CIDP with higher scores in CIDP. We also found that a cut-off UPSA score of ≥7 points has comparable AUC (0.943), sensitivity (89%), specificity (85%) and PPV (74%) in distinguishing CIDP from other neuropathies and normal controls.

In one prospective study of immune-mediated neuropathies, a novel measure was used to differentiate focal (higher intranerve CSA values) from diffuse nerve enlargement (lower intranerve CSA values) [11]. The authors reported higher internerve CSA values in immune-mediated neuropathies where disease was localised to certain nerves. Similar findings of lateralising ultrasound changes were demonstrated in multifocal motor neuropathy (MMN) (higher SSDIVA) [12]. In the current CIDP cohort, we were not able to demonstrate similar patterns of changes. This might be due to the long disease duration (mean = 2 years), which could have resulted in more homogeneous enlargement over time.

In the current study, a single case of MADSAM (multifocal acquired demyelinating sensory and motor neuropathy) or multifocal CIDP was recruited, which did not allow for meaningful comparison. In one study, MMN and MADSAM demonstrated regional nerve enlargement, whereas in CIDP, multifocal and diffuse nerve enlargement occurred in almost equal frequencies [25]. These findings suggest that the differentiation between immune-mediated neuropathies based on their homogeneity patterns of diffuse vs. focal was inaccurate. Instead, scores, such as the UPSS and its sub-score, the homogeneity score and the regional nerve enlargement index, were better tools at quantifying the extent and pattern of nerve enlargement [25].

The current study had several limitations. The number of patients in each subgroup was small, and a larger number would be helpful in determining with greater significance the utility of the UPSA score. The lower limb nerves were not included in the calculation of intranerve CSA variability, which may have affected the internerve CSA values. However, it is well-established that ultrasound studies are limited in the lower limb nerves where the nerves are deep and not easily visualised [11]. For these reasons, the UPSA scores were not calculated in six patients with CIDP due to missing data. We were also unable to generate SSDIVA for the AIDP group as the ultrasound study was performed in unilateral limbs. The inner boundaries of the nerves rather than the outer boundaries were adopted in determining nerve CSA due to the poorly defined margins of the latter on ultrasound. This meant that we could not further explore the utility of the outer nerve CSA as a disease biomarker. With improvements in ultrasound resolution, future studies to further investigate are warranted.

In conclusion, nerve ultrasound is a useful tool to facilitate the diagnosis of CIDP, especially when nerves are inexcitable on NCS. However, peripheral nerve ultrasound (i.e., UPSA) could not reliably distinguish non-CIDP demyelinating neuropathy (AIDP) from healthy controls and axonal neuropathies without also incorporating the UPSB score (scoring for cervical roots and vagus nerve) [7]. The current study suggests that quantifying the extent of nerve enlargement using the UPSA score is superior to CSA values alone in differentiating CIDP from other neuropathies.

## Figures and Tables

**Table 1:** Demographics and baseline characteristics.

Characteristics	CIDP (n = 34)	AIDP (n = 15)	Axonal (n = 16)	Controls (n = 30)	*p*-Value
Age, mean ± SD (years)	49.8 ± 17.4	54.2 ± 16.5	52.3 ± 20.5	47.0 ± 14.7	0.551
Sex (M:F)	21:13	10:5	8:8	19:11	0.779
Weight, mean ± SD (kg)	65.7 ± 10.7	62.8 ± 7.3	57.4 ± 9.7	66.2 ± 14.8	0.078
Height, mean ± SD (cm)	166.9 ± 7.8	165.7 ± 4.5	162.7 ± 7.8	164.2 ± 10.0	0.342
BMI, mean ± SD (kg/m^2^)	23.6 ± 3.6	22.8 ± 2.5	21.6 ± 2.9	24.5 ± 4.8	0.105
Disease duration, mean ± SD (weeks)	100.6 ± 116.3 ^†^	3.6 ± 2.6 ^†^	57.7 ± 82.1	NA	**0.006**
Electrodiagnostic class	32 Strongly supportive 1 Weakly supportive 1 Inexcitable	15 AIDP	8 Axonal GBS 4 ATTRv 3 DPN 1 VN	NA	

^†^ *p* = 0.004; CIDP, chronic inflammatory demyelinating polyneuropathy; AIDP, acute inflammatory demyelinating polyneuropathy; BMI, body mass index; ATTRv, hereditary transthyretin amyloidosis; DPN, diabetic polyneuropathy; VN, vasculitic neuropathy; NA, not applicable.

**Table 2:** Nerve ultrasound of CIDP and comparison with AIDP, axonal neuropathies and controls.

Nerve Ultrasound, CSA (mm^2^)	CIDP (n = 34)	AIDP (n = 15)	Axonal (n = 16)	Controls (n = 30)	ANOVA ^a^ (Between 4 Groups)
Median					
Wrist	13.3 ± 3.8	13.1 ± 4.3	11.4 ± 2.9	6.5 ± 1.1	***p *** **< 0.001**
Forearm	11.8 ± 5.9	9.3 ± 2.7	8.1 ± 1.4	5.1 ± 1.0	***p *** **< 0.001**
Elbow	17.5 ± 10.0	11.1 ± 3.2	9.3 ± 2.4	7.2 ± 1.7	***p *** **< 0.001**
Mid arm	18.6 ± 9.6	11.6 ± 3.1	10.0 ± 3.3	7.4 ± 1.5	***p *** **< 0.001**
Ulnar					
Wrist	7.6 ± 2.6	6.1 ± 1.0	5.2 ± 1.1	4.1 ± 1.0	***p *** **< 0.001**
Forearm	9.5 ± 5.5	6.3 ± 1.3	5.9 ± 1.5	4.7 ± 1.1	***p *** **< 0.001**
Elbow	12.0 ± 7.2	11.5 ± 3.7	7.6 ± 1.8	6.2 ± 1.4	***p *** **< 0.001**
Mid arm	14.0 ± 11.4	7.3 ± 2.0	7.2 ± 2.0	5.8 ± 1.8	***p *** **< 0.001**
Fibular					
Knee	15.7 ± 14.2	10.3 ± 3.3	9.5 ± 3.2	7.8 ± 2.0	***p *** **= 0.005**
Fibular head	12.0 ± 4.6	10.3 ± 2.6	9.5 ± 2.9	9.4 ± 2.2	*p* = 0.018
Tibial					
Knee	36.8 ± 16.5	31.3 ± 10.9	26.8 ± 7.1	12.5 ± 2.7	***p *** **< 0.001**
Ankle	18.2 ± 10.7	15.9 ± 4.0	14.8 ± 3.1	10.8 ± 2.0	***p *** **= 0.001**
Superficial radial	3.4 ± 2.1	2.5 ± 0.9	2.7 ± 0.9	1.1 ± 0.2	***p *** **< 0.001**
Sural	4.0 ± 2.6	2.5 ± 0.8	2.5 ± 0.7	1.4 ± 0.5	***p *** **< 0.001**
Median intranerve CSA variability	2.10 ± 1.39	1.89 ± 0.44	1.75 ± 0.35	1.59 ± 0.23	*p* = 0.140
Ulnar intranerve CSA variability	2.08 ± 1.08	2.06 ± 0.57	1.74 ± 0.32	1.74 ± 0.45	*p* = 0.199
Median SSDIVA	1.28 ± 0.26	-	1.30 ± 0.37	1.16 ± 0.17	*p* = 0.194
Ulnar SSDIVA	1.24 ± 0.20	-	1.38 ± 0.43	1.25 ± 0.23	*p* = 0.440
Internerve CSA variability	1.52 ± 0.65	1.32 ± 0.30	1.36 ± 0.36	1.26 ± 0.25	*p* = 0.159
UPS-A	9.9 ± 2.9	5.9 ± 2.0	4.6 ± 1.9	0.7 ± 1.1	***p *** **< 0.001**
UPS-A ≥ 7	25/28 (89.3%)	5/15 (33.3%)	4/16 (25.0%)	0/30 (0%)	***p *** **< 0.001**

Significant differences are highlighted in bold. ^a^ Significance is set as *p* < 0.0125 for ANOVA with comparison between groups. CSA, cross-sectional area; SSDIVA, side-to-side difference ratio of the intranerve CSA variability; UPS-A, ultrasound pattern subscore-A.

**Table 3:** Post-hoc analysis.

Nerve Ultrasound	*p*-Value ^b^ (CIDP vs. Controls)	*p*-Value ^b^ (CIDP vs. AIDP)	*p*-Value ^b^ (CIDP vs. Axonal)	*p*-Value ^b^ (AIDP vs. Controls)	*p*-Value ^b^ (AIDP vs. Axonal)	*p*-Value ^b^ (Axonal vs. Controls)
Median						
Wrist	**<0.001**	0.998	0.224	**<0.001**	0.450	**<0.001**
Forearm	**<0.001**	0.173	**0.011**	**0.004**	0.813	0.056
Elbow	**<0.001**	**0.009**	**<0.001**	0.216	0.854	0.714
Mid arm	**<0.001**	**0.002**	**<0.001**	0.142	0.885	0.528
Ulnar						
Wrist	**<0.001**	0.025	**<0.001**	**0.003**	0.576	0.143
Forearm	**<0.001**	0.022	**0.006**	0.481	0.989	0.693
Elbow	**<0.001**	0.986	0.014	**0.004**	0.105	0.799
Mid arm	**<0.001**	0.016	**0.011**	0.908	1.000	0.926
Fibular						
Knee	**0.003**	0.211	0.102	0.792	0.993	0.921
Fibular head	-	-	-	-	-	-
Tibial						
Knee	**<0.001**	0.368	0.022	**<0.001**	0.685	**<0.001**
Ankle	**<0.001**	0.696	0.370	0.092	0.973	0.230
Superficial radial	**<0.001**	0.166	0.296	**0.006**	0.990	**0.002**
Sural	**<0.001**	0.022	0.016	0.120	1.000	0.124
Median intranerve CSA variability	-	-	-	-	-	-
Ulnar intranerve CSA variability	-	-	-	-	-	-
Median SSDIVA	-	-	-	-	-	-
Ulnar SSDIVA	-	-	-	-	-	-
Internerve CSA variability	-	-	-	-	-	-
UPS-A	**<0.001**	**<0.001**	**<0.001**	**<0.001**	0.321	**<0.001**
UPS-A ≥ 7	**<0.001**	**<0.001**	**<0.001**	**0.009**	0.892	0.070

Significant differences are highlighted in bold. ^b^ Significance is set as *p* < 0.0125 (0.05/4) after Bonferroni correction for multiple comparisons. CSA, cross-sectional area; SSDIVA, side-to-side difference ratio of the intranerve CSA variability; UPS-A, ultrasound pattern subscore-A.

**Table 4:** Performance of UPS-A in differentiating CIDP from other neuropathies and controls.

UPS-A	Current Study	Grimm et al. (2015) [7]
AUC	0.943	0.950
UPS-A ≥ 7	25/28 (89.3%)	12/15 (80%)
Sensitivity	89.3%	80%
Specificity	85.2%	93%
PPV	73.5%	80%

AUC, area under the curve; UPS-A, ultrasound pattern sub-score A; PPV, positive predictive value.

## Data Availability

The data presented in this study are available on request from the corresponding author. The data are not publicly available due to confidentiality.

## Supplementary Materials

The following supporting information can be downloaded at: https://www.mdpi.com/article/10.3390/medicina59040747/s1, Table S1: The ultrasound pattern subscore-A (UPSA) for peripheral nerves; Table S2: Nerve conduction studies of patients with CIDP, AIDP and axonal neuropathies.
