# Supplementary material for: Nerve Ultrasound Score in Chronic Inflammatory Demyelinating Polyneuropathy
Source: Medicina (Kaunas). 2023 Apr 11;59(4):747. doi: 10.3390/medicina59040747 (PMC10144993; doi:10.3390/medicina59040747)
Supplement: Supplementary file 1 [file medicina-59-00747-s001.zip › medicina-2293415-supplementary.pdf]

<u>Supplementary Material</u>

Table S1. The ultrasound pattern subscore-A (UPSA) for peripheral nerves.

**Measurement**	**Boundary value (mm^2)**	**Normal<br>< 50%<br>> 50%**	**Points**
Median nerve			
Mid arm	9.9	< 9.9	0
		≥ 9.9 ≤ 14.85	1
		> 14.85	2
Elbow	9.8	< 9.8	0
		≥ 9.8 ≤ 14.7	1
		> 14.7	2
Forearm	7.2	< 7.2	0
		≥ 7.2 ≤ 10.8	1
		> 10.8	2
Ulnar nerve			
Mid arm	8.6	< 8.6	0
		≥ 8.6 ≤ 12.9	1
		> 12.9	2
Forearm	6.6	< 6.6	0
		≥ 6.6 ≤ 9.9	1
		> 9.9	2
Tibial nerve			
Popliteal	16.2	< 16.2	0
		≥ 16.2 ≤ 24.3	1
		> 24.3	2
Ankle	14.1	< 14.1	0
		≥ 14.1 ≤ 21.15	1
		> 21.15	2
Fibular nerve			
Popliteal	11.1	< 11.1	0
		≥ 11.1 ≤ 16.65	1
		> 16.65	2
Ultrasound pattern subscore-A (UPSA)			

Table S2: Nerve conduction studies of patients with CIDP, AIDP and axonal neuropathies

**Parameters**	**CIDP (n=34)**	**AIDP (n=15)**	**Axonal (n=16)**
Motor			
Median			
dCMAP (mV)	4.8 ± 3.4	5.5 ± 3.2	7.1 ± 5.0
DML (ms)	11.1 ± 6.3	9.7 ± 5.9	4.6 ± 1.9
MCV (m/s)	29.6 ± 10.7	39.2 ± 7.3	47.8 ± 9.0
Ulnar			
dCMAP (mV)	4.5 ± 2.4	4.7 ± 2.1	5.6 ± 3.7
DML (ms)	6.5 ± 3.4	5.4 ± 2.3	3.3 ± 1.2
MCV (m/s)	32.1 ± 12.8	40.2 ± 8.8	53.7 ± 8.6
Fibular			
dCMAP (mV)	1.3 ± 1.7	1.2 ± 1.0	1.8 ± 3.2
DML (ms)	11.3 ± 6.1	11.6 ± 5.6	5.4 ± 1.7
MCV (m/s)	32.9 ± 9.9	34.8 ± 7.8	38.5 ± 11.2
Tibial			
dCMAP (mV)	2.6 ± 3.0	3.2 ± 2.4	2.7 ± 3.3
DML (ms)	10.8 ± 5.3	11.4 ± 6.5	6.0 ± 2.4
MCV (m/s)	32.3 ± 8.2	31.7 ± 5.9	39.3 ± 6.8
Sensory			
Median			
SNAP (µV)	1.4 ± 3.2	1.4 ± 3.4	5.1 ± 4.9
SCV (m/s)	37.8 ± 4.4	39.0 ± 1.9	44.0 ± 8.4
Ulnar			
SNAP (µV)	1.6 ± 2.8	1.1 ± 2.3	5.0 ± 4.7
SCV (m/s)	34.9 ± 5.1	38.4 ± 6.4	46.9 ± 10.2
Superficial radial			
SNAP (µV)	9.5 ± 9.7	18.9 ± 13.4	22.3 ± 18.4
SCV (m/s)	42.6 ± 6.7	46.4 ± 7.4	53.6 ± 6.4
Sural			
SNAP (µV)	6.8 ± 8.8	7.7 ± 7.3	8.3 ± 9.9
SCV (m/s)	40.8 ± 8.3	39.6 ± 7.5	44.2 ± 5.8

dCMAP, distal compound muscle action potential; DML, distal motor latency; MCV, motor conduction velocity; SNAP, sensory nerve action potential; SCV, sensory conduction velocity
